# Identification of Functional Cell Groups in the Abducens Nucleus of Monkey and Human by Perineuronal Nets and Choline Acetyltransferase Immunolabeling

**DOI:** 10.3389/fnana.2018.00045

**Published:** 2018-06-19

**Authors:** Anja K. E. Horn, Annie Horng, Norbert Buresch, Ahmed Messoudi, Wolfgang Härtig

**Affiliations:** ^1^Anatomisches Institut, Ludwig-Maximilians Universität, München, Germany; ^2^Deutsches Schwindel- und Gleichgewichtszentrum, Ludwig-Maximilians Universität, München, Germany; ^3^RZM—Radiologisches Zentrum München-Pasing, München, Germany; ^4^Institut für Neuropathologie, Ludwig-Maximilians Universität, München, Germany; ^5^Paul-Flechsig-Institut für Hirnforschung, Universität Leipzig, Leipzig, Germany

**Keywords:** oculomotor, lateral rectus muscle, internuclear neurons, paramedian tract neurons, extracellular matrix, perineuronal nets

## Abstract

The abducens nucleus (nVI) contains several functional cell groups: motoneurons of the singly-innervated twitch muscle fibers (SIF) and those of the multiply-innervated muscle fibers (MIF) of the lateral rectus muscle (LR), internuclear neurons (INTs) projecting to the contralateral oculomotor nucleus (nIII) and paramedian tract-neurons (PMT) that receive input from premotor neurons of the oculomotor system and project to the floccular region. In monkey, these cell populations can be delineated by their chemical signature. For correlative clinico-pathological studies the identification of the homologous cell groups in the human nVI are required. In this study, we plotted the distribution of these populations in monkey nVI by combined tract-tracing and immunohistochemical staining facilitating the identification of homologous cell groups in man. Paraffin sections of two Rhesus monkeys fixed with 4% paraformaldhehyde and immunostained with antibodies directed against choline acetyltransferase (ChAT) as marker enzyme for cholinergic neurons and chondroitin sulfate proteoglycan (CSPG) to detect perineuronal nets (PNs) revealed four neuron populations in nVI with different chemical signatures: ChAT-positive and CSPG-positive SIF motoneurons, ChAT-positive, but CSPG-negative MIF motoneurons, and ChAT-negative neurons with prominent PNs that were considered as INTs. This was confirmed by combined immunofluorescence labeling of cholera toxin subunit B (CTB) or wheat germ agglutinin (WGA) and ChAT or CSPG in nVI sections from cases with tracer injections into nIII. In the rostral part of nVI and at its medial border, populations of ChAT-negative groups with weak CSPG-staining, but with strong acetylcholinesterase (AChE) activity, were identified as PMT cell groups by correlating them with the location of anterograde tracer labeling from INTs in nIII. Applying ChAT- and CSPG-immunostaining as well as AChE staining to human brainstem sections four neuron groups with the same chemical signature as those in monkey could be identified in and around the nVI in human. In conclusion, the distribution of nVI neuron populations was identified in human based on findings in monkey utilizing their markers for cholinergic neurons and their different ensheathment by PNs of the extracellular matrix.

## Introduction

Recording and anatomical tract-tracing studies in vertebrates revealed that the abducens nucleus (nVI) contains several functional cell groups (for review, Büttner-Ennever, 2006; Büttner-Ennever and Horn, 2014). In mammals, the largest population is formed by motoneurons that innervate the lateral rectus muscle (LR) of the ipsilateral eye. The extraocular muscles (EOMs) consist of different muscle fiber types that can be subdivided into two main categories based on their innervations: singly-innervated muscle fibers (SIF) that correspond to the limb muscles and respond with a twitch upon electrical stimulation and multiply-innervated muscle fibers (MIF) that respond with slow tonic contractions after stimulation. Twitch SIFs are associated with classical en plaque endings at their mid part, whereas MIFs exhibit small en grappe endings that are distributed along the whole length of the muscle fibers (Spencer and Porter, 2006). Both muscle fiber types are innervated by separate sets of motoneurons. Taking advantage of the spatial separation of the en plaque endings and en grappe endings tracer injections into the distal part of the EOM revealed that the MIF motoneurons of each EOM were localized in the periphery of the respective motonuclei (Büttner-Ennever et al., 2001). Thereby, motoneurons targeting singly-innervated twitch muscle fibers (SIF) are located within the boundaries of nVI and MIF motoneurons form a shell of scattered neurons around the medial and dorsal aspect of nVI (Büttner-Ennever et al., 2001; Eberhorn et al., 2005).

Another large neuron population within nVI is represented by the internuclear neurons (INTs) whose axons travel up within the contralateral medial longitudinal fascicle (MLF) to target the medial rectus (MR) motoneurons in the contralateral nIII (Baker and Highstein, 1975). They have been consistently found in nVI of all vertebrates with slight variations in their exact location that correlates with the placement of the eyes (for review, Evinger, 1988). Motoneurons and INTs receive afferents from the same premotor neurons in the prepositus hypoglossi nucleus, paramedian pontine reticular formation (PPRF) and medial vestibular nuclei; thereby the INTs provide the neuroanatomical basis for conjugate eye movements (for review, Büttner-Ennever, 2006; Horn and Leigh, 2011).

In monkey, another functional cell group has been identified at rostral parts of nVI, which is referred to as the paramedian tract neurons (PMT; see Büttner-Ennever and Horn, 2014). PMT neurons form a collection of separate small groups around the midline throughout the pontine and medullary reticular formation that receive inputs from premotor neurons of the oculomotor system and project to the flocculus and ventral paraflocculus (Büttner-Ennever, 1992). According to the nomenclature in monkey the PMT cell groups in the vicinity of nVI include “the intrafascicular nucleus of the preabducens area,” the supragenual nucleus (SG) and “the rostral cap” of nVI (Langer et al., 1985, 1986).

Furthermore, cell groups within nVI in monkey and rat were shown to exhibit specific histochemical properties: SIF- and MIF-motoneurons are both cholinergic, but MIF-motoneurons lack immunoreactivity for non-phosphorylated neurofilaments and chondroitin sulfate proteoglycan (CSPG)-based perineuronal nets (PNs). As SIF-motoneurons, INTs are ensheathed by PNs, but they are not cholinergic and therefore choline acetyltransferase (ChAT)-immunonegative (Eberhorn et al., 2005, 2006). The PMT cell groups around nVI are defined by their connections and cytoarchitecture (Langer et al., 1985; Büttner-Ennever et al., 1989). In order to perform correlative anatomical-clinical *post mortem* studies in human tissue, it is essential to identify the different functional cell groups in the human brainstem. So far only few studies report on the cytoarchitecture of the human nVI based on Nissl staining or the Golgi-Cox method, but without any functional correlation (Vijayashankar and Brody, 1977; Bianchi et al., 1996).

Based on the histochemical properties of SIF- and MIF-motoneurons in and around the nIII in monkey the homologous cell groups were identified in human previously (Horn et al., 2008; Che Ngwa et al., 2014; Zeeh et al., 2015). Thereby, an updated topographical map of the oculomotor nucleus has been created (Che Ngwa et al., 2014). Here we performed a similar comparative study of nVI to identify the homologous cell groups of SIF- and MIF-motoneurons, INTs and PMT neurons in human (Buresch, 2005; Horng, 2011). Accordingly, in the present study we used antibodies directed against ChAT, CSPG or aggrecan link protein (ACAN) and AChE enzyme histochemistry to delineate the functional cell groups in monkey and human by their chemical neuroanatomy.

## Materials and Methods

### Antibodies

#### Choline Acetyltransferase (ChAT)

Cholinergic motoneurons were detected with an affinity-purified polyclonal goat anti-ChAT (AB144P, Chemicon, Temecula, CA, USA) directed against the whole enzyme isolated from human placenta, which is identical to the brain enzyme (Bruce et al., 1985). In immunoblots, this antibody recognizes a 68–70 kDa protein. The appearance and distribution of ChAT-positive neurons with this antibody in the present study is identical to previous data (Eberhorn et al., 2005).

#### Chondroitin Sulfate Proteoglycan (CSPG)

PNs were routinely detected with two antibodies directed against CSPG components: (1) a mouse monoclonal antibody (clone Cat-301; MAB5284, Chemicon) directed against a brain CSPG core protein, obtained with feline spinal cord fixed gray matter as immunogen; and (2) a polyclonal rabbit antiserum (Biogenesis, 2083–5005; Poole, UK) raised against CSPG purified from bovine nasal cartilage and digested with chondroitinase ABC. It recognizes the antigenic determinants present on the sulfated glucuronic acid-*N*-acetyl-galactosamine disaccharide unmasked by chondroitinase ABC digestion (Bertolotto et al., 1986; Härtig et al., 1994; Matthews et al., 2002).

As a further CSPG marker, a monoclonal antibody raised against purified human articular cartilage aggrecan (ACAN) was used. The applied liquid tissue culture supernatant was obtained from Acris (Herford, Germany; SMI1353; clone HAG7D4; Brückner et al., 2008).

#### Wheat Germ Agglutinin (WGA)

The tracer wheat germ agglutinin (WGA; EY Labs, San Mateo, CA, USA) was detected with a polyclonal goat antibody (AS-2024; Vector, Burlingame, CA, USA).

#### Cholera Toxin Subunit B (CTB)

Polyclonal goat anti-choleragenoid (List, Campbell, CA, USA) was used to detect the tracer cholera toxin subunit B (CTB) provided by the same manufacturer. Tracing and detection method of CTB has been successfully applied in numerous previous studies (see e.g., Büttner-Ennever et al., 2001).

### Monkey

Brainstem sections of six macaque monkeys from previous anatomical projects were used in this study (Wasicky et al., 2004; Ahlfeld et al., 2011; Lienbacher et al., 2011; Table 1). Frozen sections from three cases and paraffin sections from one case were used and processed in the same way as the human case (see below). Selected sections of two monkey cases (M2, M3), who had received a tract-tracer injection with either 1% choleratoxin subunit B (CTB; List) or 2.5% WGA (EY Laboratories) into the oculomotor nucleus (nIII), which retrogradely labeled the INTs in the contralateral nVI were used from previous studies (Wasicky et al., 2004; Ahlfeld et al., 2011; Lienbacher et al., 2011). All cases had been fixed by 4% paraformaldehyde (PFA) in 0.1 M phosphate buffer (PB; pH 7.4) and subsequent immersion of the brains in 10% sucrose in 0.1 M PB and transferred to 30% sucrose prior to frozen sectioning. The brainstems were cut at 40 μm in the transverse stereotaxic plane. All experimental procedures in monkey conformed to the State and University Regulations on Laboratory Animal Care, including the Principles of Laboratory Animal Care (NIH Publication 85-23, Revised 1985), and were approved by the Animal Care Officers and Institutional Animal Care and Use Committees at Emory University and University of Washington, where all surgical interventions and perfusions were made.

**Table 1:** Rhesus monkey cases.

Case	Tract-Tracer	Fixation	Cutting	Staining for light microscopy	Staining for fluorescence microscopy
M1		4% PFA	paraffin	ChAT (DAB-Ni) + CSPG (DAB)	
M2	CTB in nIII	4% PFA	frozen	CTB	CTB + ChAT
M3	WGA in nIII	4% PFA	frozen		ACAN + WGA
M4		4% PFA	frozen	AChE enzyme histochemistry	
M5		4% PFA	frozen	ChAT, Nissl	
M6		4% PFA	frozen	CSPG,	

*List of used monkey cases providing information about experiments, fixation, sectioning and staining methods. ACAN, aggrecan; AChE, acetylcholinesterase; ChAT, choline acetyltransferase; CSPG, chondroitin sulfate proteoglycan; CTB, choleratoxin subunit B; DAB, diaminobenzidine; nIII, oculomotor nucleus; WGA, wheat germ agglutinin*.

### Human

The brainstems from three post mortem human cases (H1, H2, H3) were obtained 24 h after death through the Reference Center for Neurodegenerative Disorders of the Ludwig-Maximilians Universität, one case (H4) 8 h after death from the Department of Forensic Medicine (for details see Table 2). All procedures were approved by the Local Research Ethics Committees of the Klinikum der Ludwig-Maximilians Universität. All subjects or next of kins gave written informed consent in accordance with the ethical standards laid down in the 1964 Declaration of Helsinki. The age of the donors ranged from 57 years to 81 years, and there was no history of neurological disease. Tissue blocks of three cases were immersed in 4% PFA in 0.1 M PB, pH 7.4, for 2–6 days, one brainstem was fixed in 1% PFA and 2.5% glutaraldehyde (GA) for 12 h before cutting with a vibratome. Three brainstems (H1, H2, H3) were embedded in paraffin. Next 10- and 20 μm-thick serial sections were cut and immunostained on the slides after deparaffination and rehydrating (Table 1).

**Table 2:** Human cases.

Case	*Post-mortem* delay	Fixation	Fixation duration	Section type	Staining methods
H1	24 h	4% PFA	2 days	paraffin	ChAT (DAB-Ni) + CSPG (DAB)
H2	24 h	4% PFA	6 days	paraffin	ChAT (DAB-Ni) + CSPG (DAB)
H3	24 h	4% PFA	6 days	paraffin	Nissl
H4	8 h	1% PFA/2.5% glutaraldehyde	12 h	vibratome	AChE enzyme histochemistry

*List of used human cases providing information about post-mortem delay, fixation, sectioning and staining methods. AChE, acetylcholinesterase; ChAT, choline acetyltransferase; CSPG, chondroitin sulfate proteoglycan; CTB, choleratoxin subunit B; DAB, diaminobenzidine; DAB-Ni, nickel-enhanced DAB; WGA, wheat germ agglutinin*.

### Staining Methods

#### Combined Immunofluorescence Labeling of Tracer and ChAT or Perineuronal Nets in Monkey

At the pontomedullary junction series of equidistant sections were processed for different immunofluorescence staining combinations. For the simultaneous detection of CTB and ChAT sections of case M2 were incubated in 5% normal donkey serum in 0.1 M Tris-buffered saline (TBS), pH 7.4, containing 0.3% Triton X-100 (NDS-TBS-T) for 1 h at room temperature. Subsequently, the sections were processed with a mixture of goat anti-ChAT (1:100; Chemicon) and rabbit anti-CTB (1:5000; List) in NDS-TBS-T for 48 h at 4°C. After three washes in 0.1 M TBS, the sections were treated with a mixture of Cy3-conjugated donkey anti-rabbit IgG (1:200; Dianova) and Alexa Fluor 488-tagged donkey anti-goat IgG (1:200; Molecular Probes, Eugene, OR, USA) for 2 h at room temperature. Sections of case M3 were incubated in NDS-TBS-T and then processed with a mixture of goat anti-WGA (1:250 in NDS-TBS-T) and mouse anti-ACAN (1:75). After three washes in 0.1 M TBS, the sections were incubated with a mixture of Alexa Fluor 488-conjugated donkey anti-goat IgG (1:200; Molecular Probes) and Cy3- conjugated donkey anti-mouse IgG (1:200; Dianova). After a short rinse in distilled water sections were dried and coverslipped with permanent aqueous mounting medium (Gel/Mount; Biomeda, San Francisco, CA, USA) and stored in the dark at 4°C.

#### Immunoperoxidase Single Staining of CTB, ChAT, CSPG in Monkey

For single immunoperoxidase staining for either CTB, ChAT or CSPG selected brainstem sections were processed free-floating with polyclonal goat antibodies directed against either CTB (1:20,000; List) or ChAT (1:100; Chemicon) or with rabbit anti-CSPG (1:5000; Biogenesis) for 48 h at 4°C. All markers were visualized by binding of biotinylated donkey-anti-goat IgG (1:200; Vector Lab) or biotinylated donkey anti-rabbit IgG (1:200; Vector Lab) followed by extravidin-peroxidase (1:1000; Sigma) and diaminobenzidine (DAB) as chromogen to yield a brown color.

#### Immunoperoxidase Double Staining of ChAT and CSPG in Monkey and Human

Series of paraffin sections from monkey (M1) and human (H1, H2) were processed for the concomitant detection of ChAT-positive neurons and CSPG-containing PNs as described previously (Horn et al., 2008). After deparaffination and rehydratation the sections underwent an antigen retrieval procedure by incubation in 0.01 M sodium citrate buffer (pH 6) in a water bath at 90°C for 10 min. Subsequently, sections were washed in 0.1 M Tris-buffered saline, pH 7.4 (TBS), treated with 1% H_2_O_2_ in TBS for 30 min, were rinsed again, and preincubated with NDS-TBS-T for 1 h at room temperature. The sections were then processed with a mixture of goat anti-ChAT (1:100; Chemicon) and rabbit anti-CSPG (1:1000; Biogenesis) in NDS-TBS-T for 16 h at room temperature. After washing in 0.1 M TBS, the sections were incubated in a mixture of biotinylated donkey anti-goat IgG (1:500, Dianova) and donkey anti-rabbit IgG (1:100 Dianova) in TBS containing 2% bovine serum albumin (TBS-BSA) for 1 h. For the detection of ChAT, the sections were incubated with preformed streptavidin/biotinyl-peroxidase complexes for 1 h according to Härtig et al. (1995). Two rinses in 0.1 M TBS were followed by one wash with 0.05 M Tris-buffer, pH 8. The staining with 0.025% DAB, 0.4% ammonium nickel sulfate and 0.015% H_2_O_2_ in 0.05 M Tris-buffer, pH 8, for 10 min resulted in grayish-black ChAT-positive structures. After a thorough washing and blocking of residual peroxidase activity with 0.6% H_2_O_2_ in 0.1 M TBS, the sections were incubated in rabbit peroxidase-anti-peroxidase (1:500 in TBS-BSA; Dianova) for 1 h. After two rinses with TBS and one wash in 0.05 M Tris-buffer, pH 7.6, the sections were stained with 0.05% DAB and 0.015% H_2_O_2_ as chromogen for 10–15 min resulting in a brown staining of PNs. The sections were extensively washed with TBS, briefly, with distilled water, air-dried and coverslipped with Entellan in toluene (Merck, Darmstadt, Germany).

#### Acetylcholinesterase (AChE) Histochemistry

One monkey and one human case fixed with 1% PFA and 2.5% GA were applied to the enzyme-histochemical detection of AChE activity according to Geneser-Jensen and Blackstad (1971). In brief, after three washes in 0.25 M TBS, pH 7.4 free floating sections were incubated overnight in 100 ml of a solution containing 100 mg acetylthiocholine iodide, 7.2 mg ethopropazine hydrochloride, 75 mg glycine, 50 mg copper (II) sulfate, 410 mg sodium acetic anhydride in acetate buffer, pH 5. After three washes in 0.25 TBS for 10 min each, the sections were processed in 10% potassium hexacyanoferrate (II) solution until a brown color appeared. Finally, the sections were washed in distilled water, mounted, dried, dehydrated and coverslipped with DEPEX (Serva, Heidelberg, Germany).

### Analysis of Stained Sections

Fluorescently labeled sections were examined with a Leica microscope DMRB (Bensheim, Germany) equipped with appropriate filters for red fluorescent Cy3 (N2.1) and green fluorescent Cy2 or Alexa 488 (I3). Photographs of brightfield and fluorescence preparations were taken with a digital camera (Pixera Pro 600 ES; Klughammer, Markt Indersdorf, Germany) mounted on the microscope (Leica DMRB, Bensheim, Germany). The images were captured on a computer with Pixera Viewfinder software (Klughammer) and processed with Photoshop 7.0 software (Adobe Systems, Mountain View, CA, USA). The sharpness, contrast and brightness were adjusted to reflect the appearance of the labeling seen through the microscope. All pictures were arranged and labeled with drawing software (Coreldraw 11.0; COREL).

Paraffin sections of monkey and human nVI stained for ChAT and CSPG were imaged using a slide scanner (Mirax MIDI, Zeiss) equipped with a plan Apochromate objective (×20). The digitized images were viewed on a computer with the free software Panoramic Viewer (3DHistech; 1.152.3). Different neuron populations were plotted on zoomed images using a counter plugin (3DHistech). The images were taken as template for the reconstructions using drawing software (Coreldraw 11.0; COREL). This software was used to arrange and label the figure plates.

## Results

### Delineation of Functional Cell Groups in nVI of Monkey

**Figure 1:**
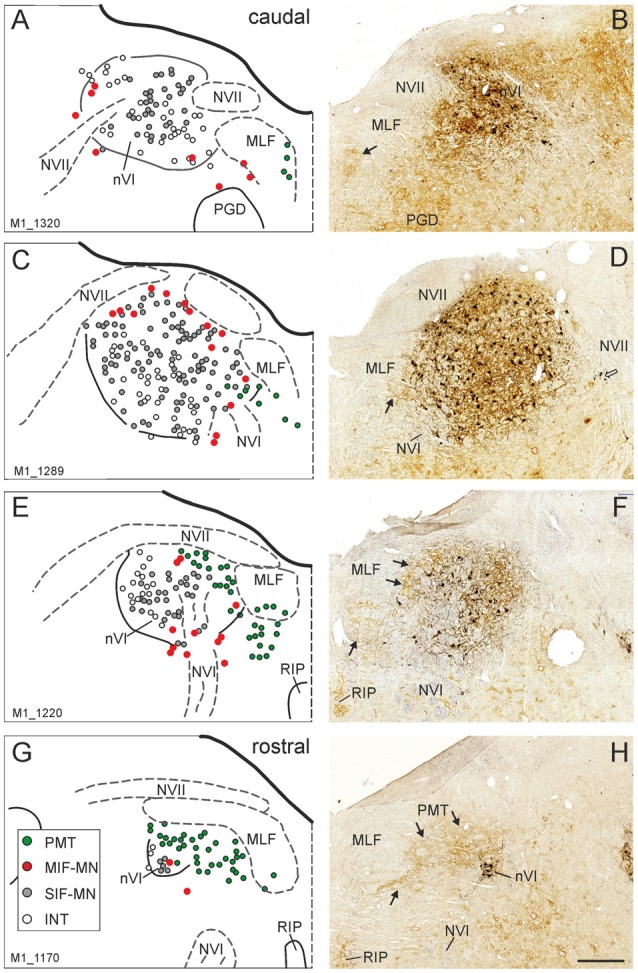
Series of transverse brainstem sections through the monkey abducens nucleus (nVI) from caudal to rostral. Immunoreactivities of choline acetyltransferase (ChAT) in black and chondroitin sulfate proteoglycan (CSPG) in brown to reveal perineuronal nets (PNs). The left column **(A,C,E,G)** shows schematic drawings of the sections presented in the right column **(B,D,F,H)**, where the functional cell groups, e.g., motoneurons of singly-innervated (SIF-MN, gray dots) and multiply-innervated muscle fibers (MIF-MN, red dots), internuclear neurons (INTs, open circles) and paramedian tract neurons (PMT; green dots) are plotted. Scale bar = 500 μm in (**H**; applies to **A–H**). MLF, medial longitudinal fascicle; NVII, facial nerve; PGD, nucleus paragigantocellularis dorsalis; RIP, nucleus raphe interpositus.

**Figure 2:**
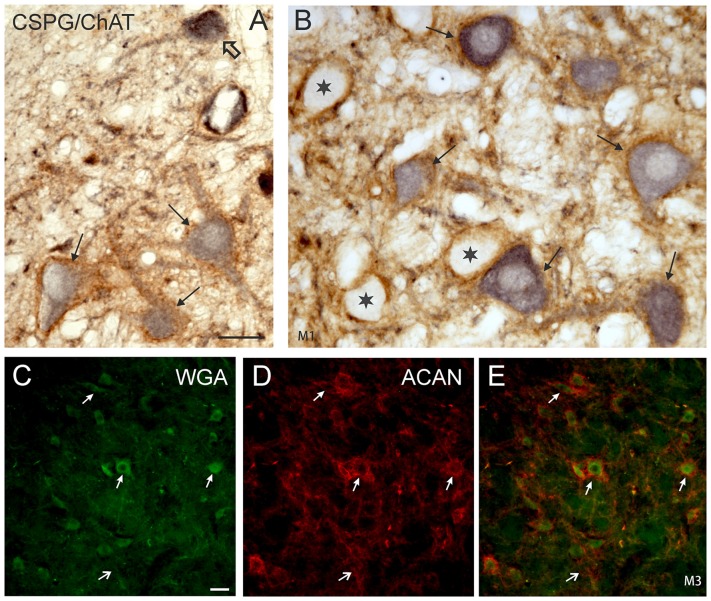
**(A,B)** Detailed microphotographs of sections from Figure 1 showing the monkey nVI stained for ChAT (black) and CSPG (brown). ChAT-positive neurons lacking PNs represent motoneurons of MIF (open arrow) **(A)**, those enwrapped by PNs represent motoneurons of singly-innervated muscle fibers (thin arrows) **(A,B)**, and the ChAT-negative ones INTs (**B**, star). **(C,D,E)** Image from double immunoflourescence in nVI of a monkey that had received a wheat germ agglutinin (WGA) injection into the oculomotor nucleus to backlabel INTs shown in **(C)** (green). Detection of aggrecan (ACAN) revealed that INTs (**C**, small arrows) are enwrapped by PNs (**D**, small arrows), more clearly seen in the overlay (**E**, arrows). The large arrow indicates a WGA-negative neuron enwrapped by a PN, which could represent a motoneuron. Scale bar = 30 μm in (**A**; applies to **A,B**); 30 μm in (**C**; applies to **C–E**).

**Figure 3:**
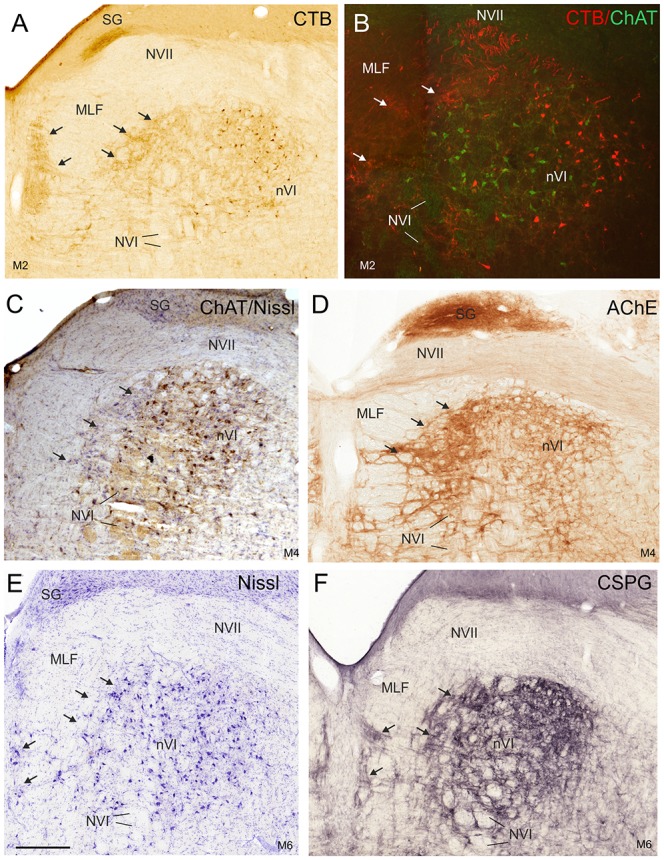
Detailed views of corresponding planes of the right nVI in transverse monkey sections from different experiments. **(A,B)** Identification of INTs *via* retrograde labeling with choleratoxin subunit B (CTB) injection into the oculomotor nucleus (nIII; **A,B**, red) and their absence of ChAT-immunoreactivity (**B**, green). Anterograde CTB-labeling from nIII highlights **(A)** the paramedian tractneurons (PMT) in the supragenual nucleus (SG) and **(B)** the “intrafascicular nucleus of the preabducens area” forming bridges between the medial nVI and midline (**A–F**; arrows). The PMT neurons are not ChAT-positive (**C**, arrows), but are highlighted by acetylcholinesterase staining (AChE) in the SG and the “intrafascicular nucleus of the preabducens area” (**D**, arrows). The appearance of PMT neurons is further demonstrated in corresponding sections stained for Nissl (**E**, arrows) and CSPG (**F**, arrows). Scale bar = 500 μm in (**E**; applies to **A–F**). MLF, medial longitudinal fascicle; NVI, abducens nerve.

The simultaneous detection of CSPG and ChAT in monkey paraffin sections revealed that the vast majority of cholinergic neurons within nVI is associated with prominent CSPG-immunoreactive PNs enwrapping somata and proximal dendrites (47%; *N*_tot_ = 545; Figures 1B,D,F,H, 2A,B, thin arrows). As described earlier a small population of cholinergic neurons devoid of PNs is present in the periphery of nVI, mainly in the medial and dorsal aspect (7.5%; Figures 1A,C,E,G, red dots; Figure 2A, open arrow). Within nVI, a large number of ChAT-negative neurons enwrapped with CSPG-based PNs were noted (26.4%), which most probably include INTs (Figure 2B, stars). This observation was verified by two monkey cases (M2, M3) with tracer injections in nIII from a previous study (Lienbacher et al., 2011), which underwent immunofluorescence staining. First, all retrogradely labeled INTs were associated with PNs (Figures 2C–E), second, all INTs were ChAT-negative (Figures 3A,B). As noted previously motoneurons and INTs are intermingled, but INTs are concentrated within a broad band of neurons running from dorsolateral to ventromedial within nVI (Büttner-Ennever et al., 1989). At rostral levels this band of INTs tends to lie laterally to the rootlets of the exiting abducens nerve (NVI; Figures 3A,B, red), whereas the ChAT-immunopositive motoneurons (Figure 3B, green) are concentrated in the medial part. As shown earlier, tracer uptake from INTs in nIII in the same experiments led to anterograde tracer labeling of the PMT cell groups (Büttner-Ennever et al., 1989). These involved the SG dorsal to the genu of the facial nerve (NVII; Figure 3A, arrow), a cell group within the MLF and adjacent PMTs forming cellular bridges with a cell group at the dorsomedial aspect of the rostral nVI (Figures 3A,B, arrows) that was designated as “interfascicular nuclei of the preabducens area” (Langer et al., 1985, 1986).

**Figure 4:**
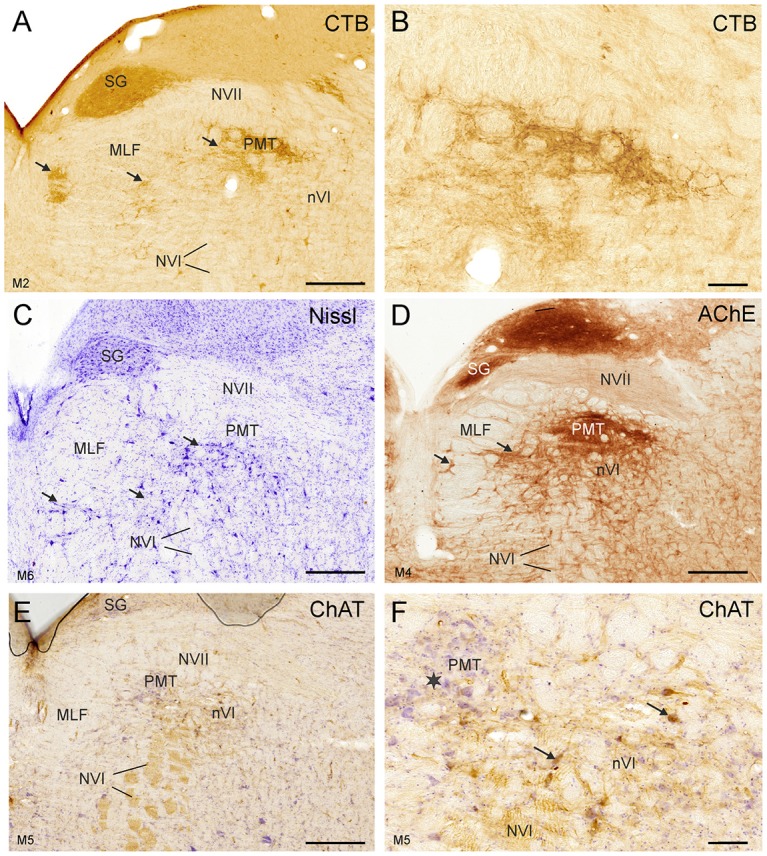
Detailed views of corresponding planes immediately rostral to the right nVI in transverse sections of monkey from different experiments. **(A,B)** Identification of PMT by anterograde choleratoxin subunit B (CTB)-labeling from the oculomotor nucleus. This includes the SG **(A)**, the “intrafascicular nucleus of the preabducens area” and the’ rostral cap of the nVI’ (**A**, arrows) shown in detail in **(B)**. The cytoarchitecture of these PMT groups is shown by Nissl staining in **(C)** (arrows). AChE staining highlights the PMT cells **(D)**, but their cell bodies are devoid of ChAT-immunoreactivity **(E,F)**, more clearly seen in the detailed view demonstrating the different morphology of ChAT-positive motoneurons (**F**, arrows) and ChAT-negative PMT neurons (star). Scale bar = 500 μm in (**E**; applies to **A,C,D,E**); 100 μm in (**B**; applies to **B,F**).

At more rostral planes another PMT cell group, the rostral cap of nVI, is outlined by anterograde labeling (Figures 4A,B). Notably, even Nissl staining revealed that all PMT cell groups show a cytoarchitecture differing from those of the nVI proper (Figures 3E, 4C). The PMT cell groups are ChAT-immunonegative (Figures 3C, 4E,F), and contain smaller, more tightly-packed neurons (Figures 3E, 4C, arrows; 4E,F), while they all display AChE-staining (Figures 3D, 4D). Diffuse CSPG-immunolabeling was present in PMT cell groups opposed to the strongly labeled distinct PNs enwrapping motoneurons and INTs in the nVI proper (1B,D,F,H, 3F, arrows).

### Abducens Nucleus in Human

**Figure 5:**
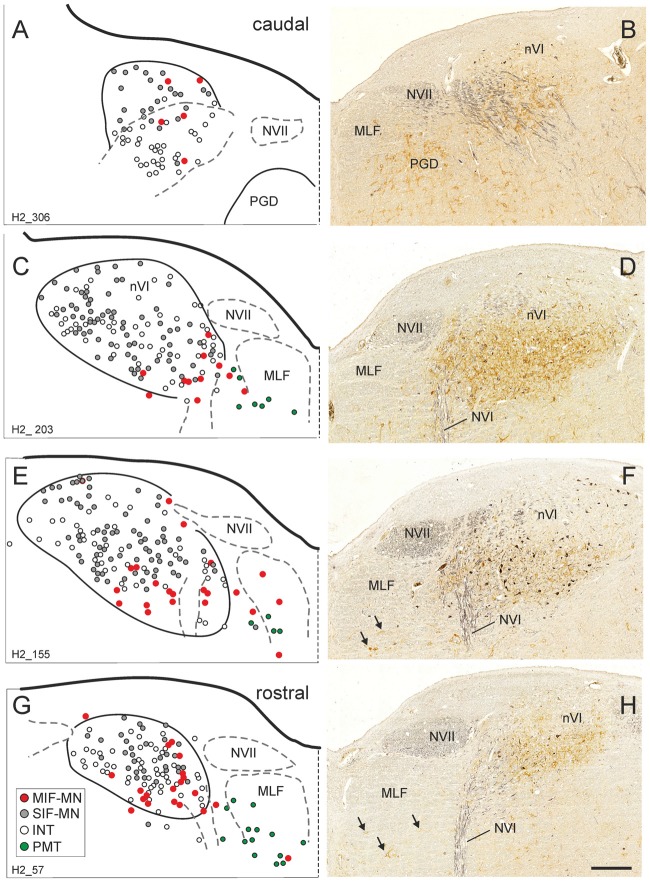
Series of transverse brainstem sections through the human nVI from caudal to rostral. Immunoreactivities for ChAT in black and CSPG in brown to reveal PNs. The left column **(A,C,E,G)** shows schematic drawings of the sections presented in the right column **(B,D,F,H)**, where the functional cell groups, e.g., motoneurons of singly-innervated (SIF-MN, gray dots) and multiply-innervated muscle fibers (MIF-MN, red dots), INTs (open circles) and PMT (green dots in **A,C,E,G** and arrows in **F,H**) are plotted. Scale bar = 500 μm in (**H**; applies to **A–H**). MLF, medial longitudinal fascicle; NVI, abducens nerve; NVII, facial nerve; PGD, nucleus paragigantocellularis dorsalis.

In human, the nVI was found on transverse sections at the pontine-medullary junction stretching between the planes of the oral end of the facial nucleus caudally and the caudal end of the motor trigeminal nucleus rostrally (Büttner-Ennever and Horn, 2014). It appears as ovoid cell group, which is bordered medially by the MLF and dorsally by the traversing fibers of the facial nerve (NVII), which form the facial genu mediodorsal to nVI (Figure 5).

### Delineation of Functional Cell Groups in nVI of Human

**Figure 6:**
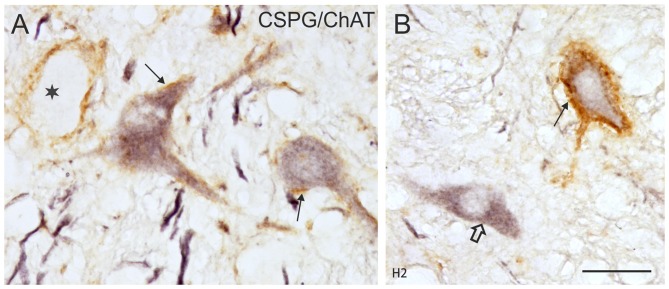
**(A,B)** Detailed microphotographs of sections from Figure 5 showing the human nVI stained for ChAT (black) and CSPG (brown). ChAT-positive neurons lacking PNs represent motoneurons of multiply-innervated muscle fibers (MIF-MN; open arrow) **(A)**, those enwrapped by PNs represent motoneurons of singly-innervated muscle fibers (**A,B**; thin arrows), and the ChAT-negative ones INTs (**B**, star). Scale bar = 30 μm in (**B**; applies to **A,B**).

As in monkey, the nVI was highlighted by its content of CSPG-immunopositive PNs (Figures 5B,D,F,H, brown) and the presence of ChAT-immunopositive neurons representing motoneurons (Figures 5B,D,F,H, black). Semiquantitative analysis revealed that about half of the ChAT-positive neurons within the boundaries of nVI are ensheathed by PNs (50.1%, *N*_tot_ = 387; Figures 6A,B, thin arrows). A small population of ChAT-positive neurons (12%)—most of them located at the medial and dorsal aspect of nVI—was devoid of PNs (Figures 5A,C,E,G, red dots; Figure 6B, open arrow). These neurons showed a similar morphology and scattered distribution as seen in monkey (compare Figures 1A,C,E,G, 5A,C,E,G). A large group of ChAT-immunonegative neurons with PNs (38%) was located within the boundaries of nVI (Figure 6A, star).

Plotting on four transversal planes through the human nVI revealed that cholinergic neurons lacking PNs were preferentially located at the ventromedial aspect of nVI (Figures 5C,E,G), whereas net-bearing cholinergic and non-cholinergic neurons were intermingled within nVI (Figures 5A,C,E,G).

**Figure 7:**
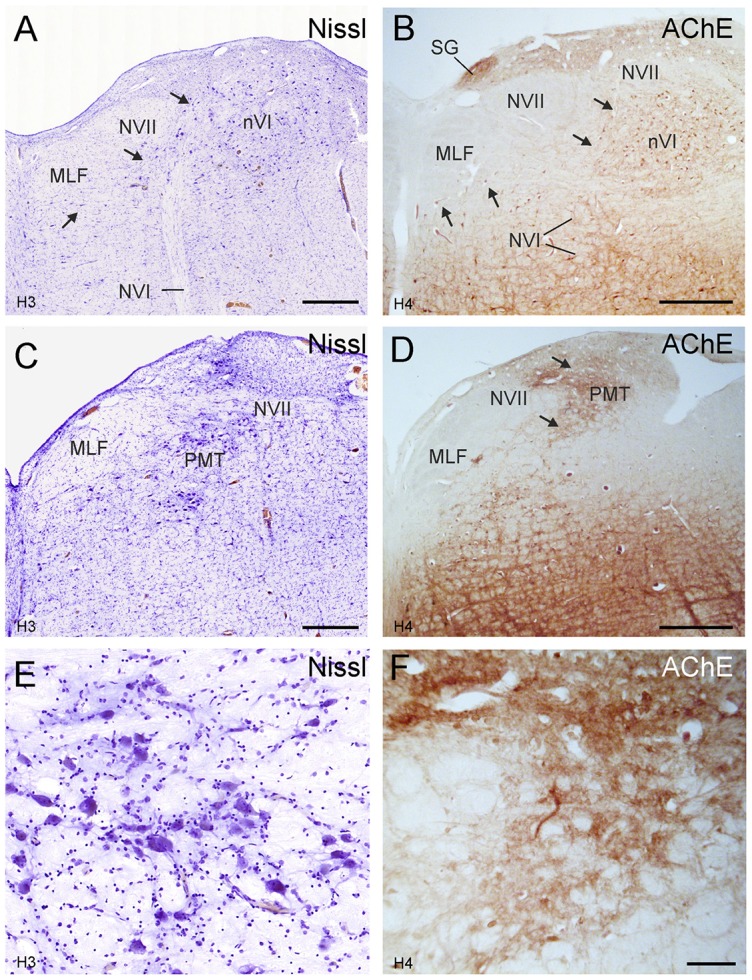
Human transverse brainstem sections at the levels of the nVI **(A,B)** and immediately rostral to nVI **(C,D)** to demonstrate the PMT. These planes correspond to those in monkey shown in Figures 3, 4, respectively. The PMT cell groups, which include the SG, the “intrafasciular neurons of the preabducens area” and “rostral cap of nVI” are high-lighted in AChE staining (**B,D** arrows), whereas their cytoarchitecture is seen in Nissl staining (**A,C**, arrows). Detailed views of the “rostral cap of nVI” are shown by Nissl and AChE staining in **(E,F)**, respectively. Scale bar = 500 μm (applies to **A–D**); 100 μm in (**F**; applies to **E,F**).

The close inspection of the nVI vicinity in AChE-stained sections revealed the putative homologous PMT cell groups in human. At planes through the rostral nVI neuronal cell bridges between neurons medial to the exiting NVI to the midline were seen with diffuse CSPG-labeling (Figures 5B,D,F,H) and AChE-staining (Figures 7A,B, arrows). On planes anterior to the rostral end of nVI a densely packed cell group appears adjacent to the MLF just underneath the traversing fibers of NVII (Figures 7C,E). This cell group shows strong AChE-staining and corresponds in location and appearance to the rostral cap of nVI in monkey (compare Figures 4C,D, 7C–F).

## Discussion

Utilizing the chemical signature of motoneurons and INTs in monkey, four functional cell groups were distinguished in and around the nVI: motoneurons of MIFs and SIFs innervating the LR, as well as INTs and PMT cell groups. Accordingly, the homologous cell groups of MIF- and SIF-motoneurons and INTs were identified in human—based on the histochemical properties found in rhesus monkey. Similarly, the putative PMT cell groups associated with nVI in human were localized.

### Identification and Location of Twitch and Non-twitch Neurons

In accordance with former studies the present work revealed two populations of cholinergic neurons in and around the nVI in monkey and man (Eberhorn et al., 2005, 2006): ChAT-positive neurons within nVI enwrapped by CSPG-positive PNs as well as ChAT-positive neurons in the dorsal and medial periphery of nVI devoid of PNs. The presence of PNs within the human nVI has previously been demonstrated with immunolabeling of ACAN, hyaluronan and proteoglycan link protein, but was not correlated with functional cell groups (Eggers et al., 2015).

Based on previous tract-tracing studies the ChAT-positive neurons with PNs are considered as motoneurons targeting twitch muscle fibers via single en plaque endplates in the LR (Büttner-Ennever et al., 2001; Eberhorn et al., 2005, 2006). This is also in accordance with the observations in nIII, where the putative twitch motoneurons within the nuclear boundaries are associated with prominent PNs in monkey and man (Eberhorn et al., 2005; Horn et al., 2008).

The ChAT-positive neurons without PNs may represent the motoneurons of MIFs and most probably include the cell bodies of palisade endings targeting the myotendinous junction of all EOMs (Lienbacher et al., 2011; Zimmermann et al., 2011). MIFs are thought to participate in gaze holding as suggested from transneuronal tracing studies in monkey, since rabies virus injections into the myotendinous junction led to transneuronal labeling only in those premotor neurons involved in gaze-holding, such as the prepositus hypoglossi nucleus or parvocellular part of the medial vestibular nucleus (McCrea and Horn, 2006; Ugolini et al., 2006). In contrast, injections of rabies virus into the muscle belly of LR targeting en grappe and en plaque endings resulted in additional transneuronal labeling of premotor neurons in the PPRF and dorsal paragigantocellular nucleus containing excitatory and inhibitory premotor burst neurons, respectively (Horn, 2006). These premotor burst neurons project monosynaptically to LR motoneurons and INTs in nVI to initiate conjugate eye movements (for review, Leigh and Zee, 2015). Unlike the circumscribed C-group of nIII, which contains the MIF neurons of the medial (MR) and inferior rectus muscle (IR), the LR MIF neurons in monkey and human do not form a compact cell group. In light of the anticipated role of MIF neurons in gaze stabilization and particularly those of the C-group in vergence as suggested by the afferent projections from brain areas involved in the near response (Wasicky et al., 2004; Bohlen et al., 2017a), it is reasonable to assume that the MIF system for the LR is less elaborated. Support comes from a comparative study on the EOM in different lateral-eyed and frontal-eyed species demonstrating that in all animals with palisade endings the MR had the highest and LR the lowest number of palisade endings (Blumer et al., 2016).

Not much is known about the location of the motoneurons of the orbital layer, which does not insert into the sclera of the eye bulb but into collagen of the pulleys and thereby alters the functional origin of the muscle, which determines its pulling direction (Demer et al., 1995; Oh et al., 2001). A quantitative study in monkey and man revealed that approximately 50% of all muscle fibers in MR and LR are part of the orbital layer (Oh et al., 2001). Further, a recent study in cat with tracer injections into the global or the orbital layer of MR did neither reveal any segregation of the motoneuron pools nor any differences in the morphology of motoneurons innervating muscle fibers of both layers (Bohlen et al., 2017b). Therefore, the ChAT-positive neurons enwrapped by PNs in the present study must be considered as the LR SIF-motoneurons of the global and orbital layer. In addition, in monkey the PN-bearing, ChAT-positive neurons in the ventral nVI may include motoneurons of the accessory LR, which in this species forms the remaining slip of the retractor bulbi muscle present in animals with a nictitating membrane (for review, Evinger, 1988). This muscle contains only singly-innervated muscle fibers with histochemical properties of twitch muscle fibers (Schnyder, 1984). This goes along with recording studies in cat demonstrating that retractor bulbi motoneurons exhibit high frequency bursts after corneal stimulation for eye retraction (Delgado-Garcia et al., 1990).

### Identification of INTs

In cat, early electron microscopic studies applying AChE staining had already demonstrated that motoneurons differ from INTs by their histochemistry. AChE staining was found associated with the endoplasmic reticulum, the soma-dendritic and axonal surface of motoneurons, but was absent from INTs (Spencer and Baker, 1986). Later tract-tracing experiments combined with ChAT-immunolabeling had shown that unlike motoneurons the INTs of monkeys are not cholinergic, but use glutamate and/or aspartate as a transmitter (Carpenter et al., 1992; Nguyen and Spencer, 1999). In correspondence to the present and previous findings in monkey it is reasonable to consider the ChAT-negative neurons enwrapped by prominent PNs in the human nVI as the INTs (Eberhorn et al., 2005).

In cat, more than 80% of the INTs but no motoneurons express the calcium-binding protein calretinin (de la Cruz et al., 1998), but only few calretinin-positive neurons were observed in the same animal species by others (Baizer and Baker, 2005). Calretinin does not serve as a suitable marker for INTs in primates, since our previous work revealed that in rhesus monkey calretinin-positive neurons are not present within the nVI proper (McCrea and Horn, 2006). Calretinin was rather found associated with premotor pathways targeting motoneurons involved in upgaze (Horn et al., 2003; Ahlfeld et al., 2011; Adamczyk et al., 2015; Zeeh et al., 2015). With 38% the size of the putative INT population within the nVI in human is larger compared to that found in rhesus monkey (26.4%), which may indicate a more elaborate system in human. The population size of INTs based on ChAT- and CSPG-immunolabeling in monkey corresponds well to that found in tract-tracing experiments (25%–30%; Steiger and Büttner-Ennever, 1978).

The presence of motoneurons and INTs is a constant feature in vertebrates throughout the evolution and provides the neuroanatomical basis for conjugate horizontal eye movements, but their location varies between species. While birds and teleosts show a high degree of segregation of INTs and motoneurons (Labandeira-Garcia et al., 1987; Cabrera et al., 1989, 1992) a less clear separation is found in rat, guinea pig and rabbit (Evinger et al., 1987; Cabrera et al., 1988; Labandeira-Garcia et al., 1989; Straka and Dieringer, 1991). In frontal-eyed species, e.g., cat and monkey, a more or less complete intermingling of motoneurons and INTs was found (Steiger and Büttner-Ennever, 1978; Büttner-Ennever and Akert, 1981). This is confirmed for monkey and expanded to the human nVI, in the present study. In addition single cell reconstructions in cat and monkey revealed that the dendrites of motoneurons and INTs remain largely confined to the boundaries of nVI and occupy completely overlapping territories (cat: Highstein et al., 1982; squirrel monkey: McCrea et al., 1986). This strengthens the concept that inputs from premotor areas, e.g., secondary vestibular neurons, PPRF, to the nVI target both, motoneurons and INTs enabling precise conjugate eye movements in frontal-eyed species with large binocular visual fields (Langer et al., 1986; Büttner-Ennever, 2006).

### Identification of Putative PMT Cell Group Neurons

According to data on PMT neurons from Büttner-Ennever et al. (1989), anterograde tracer labeling from the INTs of nIII in monkey outlined PMT cell groups in the vicinity of nVI in the present study. Originally, the PMT cell groups around nVI had been defined by their projection targets to the flocculus in different species (for review, Büttner-Ennever, 1992). In line with the nomenclature for monkeys by Langer et al. (1985, 1986), the labeled PMT cell groups of the present study correspond to the “interfascicular nuclei of the preabducens area,” the SG and the “rostral cap of nVI”. Despite their shared histochemical properties with INTs, e.g., lack of ChAT, but association with diffuse PNs, PMT neurons can be distinguished by their cytoarchitecture, location at the rostral and medial border of nVI and strong AChE activity.

The observation of Rodella et al. (1996), who found that at least 50% of PMT neurons in rat are cholinergic, was not confirmed in the present study of monkey and human. Although the PMT cell groups were high-lighted by AChE staining (see also Buresch, 2005; Horn, 2006), this enzyme-histochemical method is not selective for cholinergic neurons as indicated by double labeling experiments, where all ChAT-immunopositive neurons are stained for AChE activity, but this does not apply *vice versa* (Armstrong et al., 1983; Eckenstein and Sofroniew, 1983; Mesulam et al., 1984). A clear example for that is apparent from the saccadic omnipause neurons in the nucleus raphe interpositus, which express strong AChE-activity also seen in our sections (not shown), but they are known to use glycine as a transmitter (Horn et al., 1994). There is now agreement that the cholinergic phenotype of a neuron is defined by the co-expression of ChAT and the vesicular acetylcholine transporter (VAChT), which are contained within the same gene locus defining a “cholinergic operon” (Erickson et al., 1994). Based on its association with synaptic vesicles VAChT-immunolabeling is more suited to identify cholinergic terminals, whereas the cytosolic ChAT identifies more clearly cholinergic cell bodies (Schäfer et al., 1995; Weihe et al., 1996). Therefore the lack of ChAT-immunostaining in their cell bodies classifies the PMT neurons as non-cholinergic. Irrespective of the definite transmitter being used the enzyme-histochemical AChE staining serves as a reliable marker to highlight PMT neurons, which were previously also found by the visualization of cytochrome oxidase activity (Buresch, 2005; Horn, 2006).

Putative PMT neurons around nVI exhibit a burst-tonic firing (Nakao et al., 1980), which might enable motor-like feedback signals to the flocculus to contribute to gaze holding. Such conclusion is indicated by pharmacological lesion experiments in monkey that lead to gaze-holding impairment mainly for vertical eye movements (Nakamagoe et al., 2000). Similarily, patients with small ponto-medullary infarctions showed a gaze-evoked nystagmus, either only in the vertical, or vertical and horizontal direction possibly caused by lesion of PMT neurons (Anagnostou et al., 2009; Lee et al., 2012; Zhao et al., 2015). Based on anatomical and lesion studies data modeling supported a function of the PMT cell groups in gaze holding *via* their connections to the cerebellum (Dean and Porrill, 2008).

### Significance of Perineuronal Nets Enwrapping MIF-MN, INTs and PMT Cells

PNs appear to play a role in different functions, which include synaptic stabilization during development, control of plasticity, ion homeostasis and neuroprotection (Soleman et al., 2013; Suttkus et al., 2016). One finding is that PNs are not associated with modulatory neurons, such as the serotonergic raphe nuclei (Hobohm et al., 1998) or the urocortin-immunopositive non-preganglionic Edinger-Westphal nucleus (Horn et al., 2008), but with highly active fast-firing neurons (Dityatev et al., 2007). Thereby the presence of PNs is often associated with the content of the calcium-binding protein parvalbumin in fast-firing neurons co-expressing the voltage-dependent potassium channel subunit Kv3.1b (Härtig et al., 1994, 1999). Accordingly, several functional cell groups of the oculomotor system that exhibit high firing rates were shown to be parvalbumin-positive and be enwrapped by prominent PNs in primates including man. This includes premotor saccadic burst neurons, omnipause neurons and motoneurons of EOM (Horn et al., 2003, 2008). With the INTs in nVI we add another functional cell group in human, which show similar firing characteristics of motoneurons (McCrea et al., 1986; Fuchs et al., 1988). As for the EOM supplied from nIII also those peripheral motoneurons of nVI target non-twitch tonic MIFs lacking PNs. Up to date there are no recording studies describing the firing characteristics, but a more tonic discharge pattern is expected. Interestingly, the MIFs of the global layer—unlike the twitch fibers—appear to be affected in amyotrophic lateral sclerosis (Tjust et al., 2017). In how far the motoneurons of the MIFs are degenerated in amyotrophic lateral sclerosis has not been shown, yet. They are devoid of PNs and their selective degeneration would support a function of PNs in neuroprotection (Suttkus et al., 2016).

In conclusion, within the nVI of monkey and man, the well-known LR motoneurons and INTs can be easily delineated from each other by their different neurotransmitters. The motoneurons of singly-innervated twitch muscle fibers and multiply-innervated non-twitch muscle fibers subserving different tasks in oculomotor control can be identified by their present or lacking ensheathment by PNs. This allows for a specific analysis of different motoneuron types in *post mortem* cases of ALS. The cytoarchitectural boundaries of the rostral nVI enclose the PMT neurons as another functional cell group that project to the flocculus and may contribute to the cerebellar control of gaze holding (Dean and Porrill, 2008). Thereby, for any analysis of projections or lesions in nVI it is essential to identify the actual functional cell group for correct interpretation of the observations, as shown for the projections of premotor neurons of the vertical saccadic system to nVI, which was found to target PMT cell groups and not motoneurons or INTs (Büttner-Ennever et al., 1989).

## Author Contributions

AHorn, AHorng and NB contributed conception and design of the study. AHorng, AM, NB and WH created the data. AHorn and WH wrote the first draft of the manuscript. All authors contributed to manuscript revision, read and approved the submitted version.

## Conflict of Interest Statement

The authors declare that the research was conducted in the absence of any commercial or financial relationships that could be construed as a potential conflict of interest.

## Abbreviations

ACAN: aggrecan
AChE: acetylcholinesterase
ChAT: choline acetyltransferase
CSPG: chondroitin sulfate proteoglycan
CTB: choleratoxin subunit B
DAB: diaminobenzidine
EOM: extraocular muscle
INTs: internuclear neurons
IR: inferior rectus muscle
LR: lateral rectus muscle
MIF: multiply-innervated muscle fiber
MLF: medial longitudinal fascicle
MR: medial rectus muscle
NDS-T: 5% normal donkey serum containing 0.3% Triton X-100
nIII: oculomotor nucleus
NVI: abducens nerve
nVI: abducens nucleus
NVII: facial nerve
PB: phosphate buffer
PFA: paraformaldehyde
PMT: paramedian tracts
PNs: perineuronal nets
PPRF: paramedian pontine reticular formation
SG: supragenual nucleus
SIF: singly-innervated muscle fiber
TBS: Tris-buffered saline
TBS-BSA: TBS containing 2% bovine serum albumin
WGA: wheat germ agglutinin.
